# LncRNA *Snhg12*/IGFBP3 axis is involved in liver fibrosis by promoting the proliferation and activation of mouse hepatic stellate cells

**DOI:** 10.1002/ccs3.12033

**Published:** 2024-05-28

**Authors:** Jingmao Liao, Qi Yuan, Lidan Luo, Xiaoxuan Hu, Zhengzheng Li, Zheng Zhang

**Affiliations:** ^1^ Department of Hepatology Hunan Provincial People's Hospital The First Affiliated Hospital of Hunan Normal University Changsha Hunan China; ^2^ Department of Vascular Surgery Hainan Provincial People's Hospital Hainan Medical College Affiliated Hainan Hospital Haikou Hainan China

**Keywords:** IGFBP3, liver fibrosis, LncRNA *Snhg12*, mouse hepatic stellate cells (mHSCs), protein stability

## Abstract

Liver fibrosis is a persistent damage repair response triggered by various injury factors, which leads to an abnormal accumulation of extracellular matrix within liver tissue samples. The current clinical treatment of liver fibrosis is currently ineffective; therefore, elucidating the mechanism of liver fibrogenesis is of significant importance. Herein, the function and related mechanisms of lncRNA *Snhg12* within hepatic fibrosis were investigated. *Snhg12* expression was shown to be increased in mouse hepatic fibrotic tissue samples, and *Snhg12* knockdown suppressed hepatic pathological injury and down‐regulated the expression levels of fibrosis‐associated proteins. Mechanistically, *Snhg12* played a role in the early activation of mouse hepatic stellate cells (mHSCs) based on bioinformatics analysis, and *Snhg12* was positively correlated with Igfbp3 expression. Further experimental results demonstrated that *Snhg12* knockdown impeded mHSCs proliferation and activation and also downregulated the protein expression of Igfbp3. *Snhg12* could interact with IGFBP3 and boost its protein stability, and overexpression of *Igfbp3* partially reversed the inhibition of mHSCsproliferation and activation by the knockdown of *Snhg12*. In conclusion, LncRNA *Snhg12* mediates liver fibrosis by targeting IGFBP3 and promoting its protein stability, thereby promoting mHSC proliferation and activation. *Snhg12* has been identified as an underlying target for treating liver fibrosis.

## Graphical abstract

“lnc Snhg12/IGFBP3 axis promote liver fibrosis by promoting HSC proliferation and activation”.

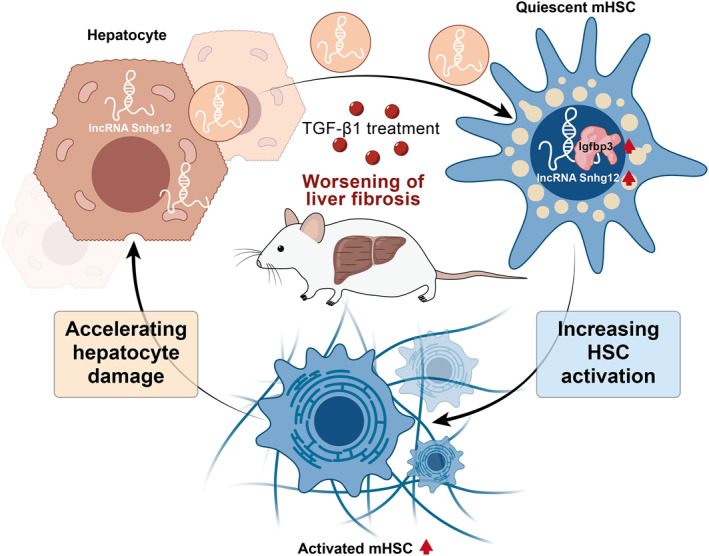

## 1 INTRODUCTION

Liver fibrosis is defined as liver damage caused by the stimulation of various persistent damage factors, such as alcoholism, cholestasis, etc..^1^ These persistent factors cause chronic inflammation in the liver, leading to hepatocyte damage and death. In order to repair damaged liver tissues, the body initiates liver regeneration and repair processes; however, in cases where the liver is stimulated by persistent injury, the repair process becomes abnormal, leading to excessive collagen deposition,^2^ fibrotic tissue proliferation, disruption of the hepatic lobular structure, and replacement of normal liver tissue with fibrotic tissue. This structural change affects the normal functioning of the liver and could also lead to the development of cirrhosis. Understanding the mechanisms of liver fibrosis and finding appropriate treatments are crucial in the prevention and management of liver disease.

The post‐genomics era has entailed a boom in transcriptomics and proteomics, thereby revealing that noncoding RNA (ncRNA), a class of RNA molecules that are not translated into protein, exerts a crucial effect on regulating gene expression and functions.^3^ Small nucleolar RNA host gene 12 (*SNHG12*), also referred to as ASLNC04080 is a novel lncRNA located on chromosome 1p35.3 that was initially found to be increased within endometrial carcinoma.^4^ Current articles on this lncRNA have focused on malignancies. For instance, *SNHG12* has been demonstrated to enhance cancer cell proliferation, migration, and invasion,^5^ and mediate resistance to chemotherapy in glioblastoma and renal cell carcinoma^5^, ^6^ and immune escape within non‐small‐cell lung cancer.^7^ Herein, *SNHG12* expression was shown to be increased within human liver fibrosis tissue samples by bioinformatics analysis. Since there are no reports on the association of *SNHG12* with liver fibrosis, their function and mechanism in liver fibrosis need to be elucidated.

Insulin‐like growth factors (IGFs), members of the insulin superfamily of growth‐promoting peptides, play vital roles in endocrine, autoimmune, and tumor diseases.^8^ IGFBP3 is the most abundant member of the family, located upon the short arm of chromosome 7. The IGFBP3 protein consists of 264 amino acids and has a molecular weight of approximately 28.7 kDa.^9^ In this study, the expression level of *Igfbp3* was shown to be increased within mouse liver fibrosis tissues by bioinformatics analysis and positively correlated with *Snhg12* expression. As previously reported, the migration and proliferation ability of cardiac fibroblasts can be suppressed by the knockdown of *IGFBP3*, which can be a potential therapeutic target for myocardial fibrosis.^10^ Therefore, *Igfbp3* was selected in our study to further elucidate its role in mouse liver fibrosis.

Herein, *Snhg12* expression was shown to be increased within mouse liver fibrosis tissue samples, and the knockdown of *Snhg12* impeded the progression of liver fibrosis. Mechanistically, *Snhg12* could target IGFBP3 and boost the protein stability of the latter, which promoted the proliferation and activation of mouse hepatic stellate cells (mHSCs) and ultimately mediated the development of hepatic fibrogenesis. LncRNA *Snhg12* is expected to be a potential target for the treatment of liver fibrosis.

## 2 MATERIALS AND METHODS

### 2.1 Bioinformatics analysis

The GSE datasets were obtained from the Gene Expression Omnibus (GEO) database (https://www.ncbi.nlm.nih.gov/geo/). Among them, GSE28619 included hepatic gene expression profiles from human alcoholic hepatitis patients (*n* = 15) and normal livers (*n* = 7)^11^; GSE84044 included liver biopsy samples from 124 chronic hepatitis B patients^12^; among them, differential lncRNAs between no fibrosis (*n* = 37) and grade 3–4 severe fibrosis (*n* = 20) were compared. Differential gene analysis was performed on this dataset using the R language limma package, setting the screening parameters to |logFC| >0.8 and ADJUSTED P.val <0.05. Plots such as volcano plots, heat maps, and gene expression profiles were performed with the ggplot2 package for the R language. GSE132662 included hepatic stellate cell samples isolated from 4 healthy mice and early fibrotic liver mice (3 weeks CCl_4_ treatment).^13^ GSE132662 was downloaded, and single‐cell RNA sequencing data were loaded using Seurat 4.1.0 and the quality was controlled and merged. The data were standardized and normalized. Highly variable genes were obtained for principal component analysis and batch effect removal. The cell population was visualized by cluster analysis and t‐distributed stochastic neighbor embedding (TSNE) dimensionality reduction. The FindAllMarkers function was subsequently used to find differentially expressed genes (DEGs) and perform cell type annotation. The FindMarkers function was used to find DEGs between groups, screen for DEGs, perform functional enrichment analysis and visualize the results. Finally, the Monocle2 was used to reconstruct cell differentiation trajectories.

GSE141821 contained liver transcriptome sequencing expression datasets from 8 cases of C57BL/6 mice and 9 cases of CCL_4_‐induced liver injury. GSE173920 contained transcriptome sequencing expression datasets from different time points of mouse HSC (mHSC) activation (*n* = 2 or 3),^14^ used for *Snhg12* expression validation. GSE151251 contained 3 cases of human HSCs and 3 cases of TGF‐β‐stimulated HSC RNA sequencing dataset for *SNHG12* expression validation.

GSE123932 contained lncRNA and mRNA expression microarray data of 6 human healthy liver tissues and 6 HBV cirrhotic tissue samples, and GSE207857 contained RNA sequencing expression data of 6 normal mouse liver tissues and 6 CCl_4_‐induced liver fibrosis mouse liver tissues,^15^ which were used for *Snhg12* and *Igfbp3* expression correlation analysis.

### 2.2 Establishment of a mouse model of liver fibrosis

Twenty five male C57BL/6 mice (8 weeks old) were procured from Hunan Silaike Jingda laboratory animal company (Changsha, China) and were modeled after 7 days of acclimatization. Mice with liver fibrosis were injected intraperitoneally with 20% CCl_4_ corn oil solution (0.05 mL/10 g) twice a week for a period of six weeks, while sham‐operated mice were subjected to injection using an equal amount of olive oil solution in the same manner.^16^ 2 weeks following the last injection with CCl_4_, mice were fasted for 12 h and had free access to water; after anesthesia, blood samples were collected, mouse livers were harvested, and then transferred to −80°C for storage or fixed in formalin for histological investigation.

The lentiviral vectors encoding short hairpin (sh)‐RNA for stable knockdown of *Snhg12* (lv‐sh‐*Snhg12*#1 and lv‐sh‐*Snhg12*#2) were designed and synthesized by Ori‐Bio (Changsha, China). 25 mice were randomly allocated into 5 groups: Sham, CCl_4_, CCl_4_ + Lv‐sh‐NC, CCl_4_ + Lv‐sh‐Snhg12#1, and CCl_4_ + Lv‐sh‐*Snhg12*#2. Control mice were injected with corn oil as a vehicle. Mice from the latter 3 groups were given 1 × 10^7^ TU of Lv‐sh‐NC, sh‐*Snhg12*#1, or sh‐*Snhg12*#2 per mouse. Mice were subjected to intravenous injection with 100 μL of Lv‐shCtrl, sh‐*Snhg12*#1, or sh‐*Snhg12*#2 lentivirus (1 × 10^7^ TU) via their tail vein twice (at week 0, 2). The ShRNA sequence of lncRNA *Snhg12* was exhibited in Table S1 in Supporting Information S1. Two weeks after the last CCl_4_ injection, the mice were sacrificed under anesthesia, and the blood sample and liver were harvested for further analysis. All experiments involving animals were approved by the Animal Care and Use Committee of Hunan Provincial People's Hospital.

### 2.3 mHSCs culture and treatment

mHSCs were obtained from ScienCell (Cat. #M5300; Carlsbad, USA) and cultivated within Stellate Cell Medium (Cat. #5301; ScienCell). For mHSCs activation, 25 ng/mL mouse TGF‐β1 (CUASBIO, Wuhan, China) was used to treat mHSCs for 0 h, 6 h, 12 h, 24 h, 48 h, and 96 h.

sh‐*Snhg12*#1 or sh‐*Snhg12*#2 vector (pLVX‐shRNA2) was used to transfect cells to achieve *Snhg12* knockdown within mHSCs. For *Snhg12* overexpression, the whole sequence of *Snhg12* was cloned into an expression vector (pLVX‐IRES‐puro) and then transfected into mHSCs. For *Igfbp3* knockdown, the sh‐*Igfbp3* vector (Ori‐Bio) was transfected into mHSCs. Lipofectamine 3000 Reagent (Thermo Fisher Scientific, Waltham, USA) was applied to implement transfection steps as directed by the manufacturer. Upon 48‐h transfection, the transfection efficiency was evaluated. For TGF‐β1 stimulation, mHSCs upon 24h‐transfection were further activated by TGF‐β1 for 24 h. The sequences used are displayed in Table S1 in Supporting Information S1.

### 2.4 Quantitative reverse transcription polymerase chain reaction (qRT‐PCR)

Samples of mHSCs and mouse liver tissues were harvested, and TRIzol (Invitrogen, Carlsbad, USA) was used to extract the total RNA. A reverse transcription kit (TaKaRa, Tokyo, Japan) was used as directed by the kit's protocol to reverse transcribe cDNA from RNA. A LightCycler 480 (Roche Diagnostics, Indianapolis, USA) fluorescent quantitative polymerase chain reaction (PCR) instrument was applied to assess gene expression, and the fluorescent quantitative PCR kit (SYBR Green Mix, Roche Diagnostics) was used as directed by the kit's protocol to perform reaction conditions. The PCR temperature cycling conditions were as follows: initial denaturation at 95°C for 10 s; 45 cycles of denaturation at 95°C for 5 s, annealing at 60°C for 10 s, and elongation at 72°C for 10s. The final cycle was followed by an extension at 72°C for 5 min. Each quantitative PCR reaction was carried out thrice. Glyceraldehyde 3‐phosphate dehydrogenase was used for the normalization of the relative expression of target genes, which was calculated using the 2^−ΔΔCt^ method, ΔΔCt = experimental group (Ct target gene—Ct internal reference)—control group (Ct target gene—Ct internal reference). The primer sequences of all genes and their internal controls are listed in Table S1 in Supporting Information S1.

### 2.5 Western blot

Total proteins were extracted from mouse liver tissue sand mHSCs by lysing the cells with radioimmunoprecipitation assay lysis solution (Beyotime, Shanghai, China). The bicinchoninic acid kit (Beyotime) was used to measure the protein content. After adding the corresponding protein volume into the sampling buffer (Beyotime) and mixing thoroughly, a 5‐min heating process in a boiling water bath was carried out to denature the protein. Electrophoresis was carried out using an initial constant voltage of 80 V for 30 min to concentrate the sample; after the voltage was switched to 120 V for 1–2 h until the bromophenol blue indicator reached the bottom edge, and electrophoresis was stopped. The transfer was carried out in an ice bath with a transfer current of 220 mA for 120 min. Subsequently, after 1–2 min washing, membranes were blocked at room temperature (RT) for 60 min and incubated at 4°C overnight using the primary antibody and then at RT for 1 h using a secondary antibody (horseradish peroxidase‐conjugated goat anti‐rabbit or anti‐mouse IgG, 1: 5000, Beijing ComWin Biotech Co., Ltd., Beijing, China). A chemiluminescent imaging system (Bio‐Rad, Hercules, USA) was used to perform detection after a drop of the developer to the membrane. Antibodies used for western blot are listed in Table S2 in Supporting Information S1.

### 2.6 Measurement of serum levels of ALT and AST

The alanine aminotransferase (ALT) and aspartate aminotransferase (AST) levels in mouse serum were determined using the ALT (E‐BC‐K235‐M, Elabscience, Wuhan, China) and AST (E‐BC‐K236‐M, Elabscience) kits as per the requirements of the manufacturer. The collected mouse blood specimens were left at RT for 30 min, followed by 10‐min centrifugation at 2000 rpm, and the supernatant, that is, serum, was taken for ALT and AST assays.

### 2.7 Determination of hydroxyproline

The Hydroxyproline Content Assay Kit (E‐BC‐K062‐M, Elabscience) was used as directed by the manufacturer to measure hydroxyproline levels within mouse liver tissues. In short, 100 mg of liver tissue was hydrolyzed within 1 mL of lysis buffer at 100°C for 5 h and collected the supernant for incubation with oxidant working solution and chromogenic working solution at 65°C for 15 min. A microplate reader (558 nm) was applied to assess the absorbance, and the hydroxyproline content was calculated using the standard curve.

### 2.8 Hematoxylin and eosin (HE) staining

After being dewaxed with xylene, paraffin‐embedded slices were subjected to rehydration with increasing concentrations of alcohol, washed with phosphate buffer saline (PBS), and subjected to 10‐min immersion in hematoxylin staining solution. The slices were subsequently subjected to 5‐s immersion in hydrochloric acid alcohol after tap water immersion, which was immersed in eosin for 2 min after a tap water immersion. The slides were subsequently sequentially subjected to 10‐s immersion in 70%, 80%, and 90% ethanol and 10‐s immersion in anhydrous ethanol, cleared with xylene for 10 min, and blocked with neutral resin. The microscope was used to capture pathological alterations within the liver tissue samples of mice.^17^

### 2.9 Sirius Red staining

After being regularly deparaffinized and hydrated, paraffin sections were subjected to 3‐min staining with hematoxylin and then 1‐h staining at RT with Sirius red staining solution (Beijing Solarbio Science and Technology Co., Ltd., Beijing, China). The sections were dehydrated using an alcohol gradient and blocked after immersion in xylene solution. Lastly, sections were observed and photographed under a microscope.

### 2.10 Masson staining

After being deparaffinized by xylene, paraffin sections were rehydrated with increasing concentrations of ethanol, washed with PBS, and stained with Weigert's ferric hematoxylin staining solution, a 1:1 mixture, for 10 min. The sections were subsequently subjected to 5‐15‐s differentiation using acidic alcohol differentiation solution, 3‐min staining using Masson bluing solution, 5‐10‐min staining using Ponceau S staining solution, and 1‐min rinsing with a weak‐acid working solution. Next, sections were subjected to 1‐2‐min washing in phosphomolybdic acid solution, 1‐min rinsing in weak‐acid working solution, 1‐2‐min staining with aniline blue staining solution, and 1‐min washing in weak‐acid working solution. Moreover, the sections were reacted with 95% and anhydrous ethanol, respectively, cleared with xylene, and blocked with neutral resin.

### 2.11 Cell counting kit (CCK)‐8 assay

The Cell Counting Kit‐8 (Beyotime) was used as directed by the manufacturer to evaluate cell viability. Single‐cell suspensions of differently transfected cells were prepared, counted, and then coated in 96‐well plates at 5000 cells per well, with 100 μL of culture medium per well. Under TGF‐β1 stimulation, 10 μL of CCK‐8 solution was added to each well following 0, 24, 48 and 72 h of incubation, and then incubated at 37°C for 2 h. A microplate reader (Bio‐Rad) was then applied to measure the absorbance at 450 nm.^18^

### 2.12 EdU staining

EdU working solution (Ribobio) was diluted to 50 μM with culture medium, followed by continued incubation of the cells for 2 h. Subsequently, after discarding the culture media, 1 mL of 4% paraformaldehyde was supplemented and fixed for 15 min at RT. The cells were then rinsed thrice with 1 mL of PBS per well, each time for 3–5 min. Subsequently, cells were incubated for 10 min at RT with 0.5% Triton X‐100. Apollo Reaction Buffer was supplemented to the wells, followed by a 30‐min incubation at RT in light‐deprived conditions. Nuclei were stained with Hoechst33342 and kept in light‐deprived conditions for 10 min at RT, and cells were rinsed thrice with a washing solution, each time for 3–5 min. The proliferating cells were observed to be red‐fluorescent and the nuclei were blue‐fluorescent under the fluorescence inverted microscope.^19^

### 2.13 Immunofluorescence staining

The transfected mHSCs were made into a cell suspension, counted at a cell density of 1 × 10^5^/mL, and inoculated onto cell slides in 24‐well plates, and immunofluorescence staining was performed after cells were adhered onto the wall for 12 h and subjected to a 24‐h stimulation with TGF‐β1. In short, after fixing cells at RT for 30 min with 4% paraformaldehyde solution, rinsed thrice in PBS, added with 0.1% TritonX‐100 solution for 20 min at RT, and blocked for 30 min at RT using 10% goat serum. Next, cells were subjected to an overnight incubation with primary antibodies (α‐smooth muscle action [α‐SMA] [55135‐1‐AP, Proteintech] and collagen I [66761‐1‐Ig, Proteintech]) diluted with 10% goat serum (Zhongshan Golden Bridge, Beijing, China) in a refrigerator at 4°C in light‐deprived conditions, rinsed thrice with PBS, and dried using filter papers. After that, cells were subjected to 1‐h incubation at 37°C with Alexa 488 or 647 labeled fluorescent secondary antibody diluted with 10% goat serum. After the addition of 4',6‐diamidino‐2‐phenylindole staining solution (Beyotime) for coloring at RT for 1 min, the cells were blocked using an anti‐fluorescence quencher and captured under a fluorescence‐inverted microscope (Olympus, Tokyo, Japan).^20^

### 2.14 RNA‐binding protein immunoprecipitation (RIP) assay

RIP detection was carried out using the Magna RIP kit (Meck Millipore, Billerica, USA). Referring to the instructions, the magnetic beads were mixed with 5 μg of anti‐rabbit IgG or 5 μg of anti‐IGFBP3 antibody, and then cell lysates and RIP immunoprecipitation buffer were incubated with the magnetic bead‐antibody complexes at 4°C in a rotary incubator overnight. After proteinase K treatment, RNA was eluted from the immunoprecipitation complex and further purified for qRT‐PCR. Primer sequences are shown in Table S2 in Supporting Information S1.

### 2.15 Protein stability assay

Cells were treated with the protein synthesis inhibitor cycloheximide (CHX) using a final concentration of 50 μg/mL. After incubating for 2, 6 and 12 h, and at the end of the incubation, changes in the half‐life of IGFBP3 protein were detected using western blot.^21^

### 2.16 RNA pull‐down assay

The full‐length *Snhg12* (sense) and anti‐sense RNA were generated with TranscriptAid T7 High Yield Transcription Kit (#K0441, Thermo Fisher Scientific) by in vitro transfection of vectors. Afterward, PierceTM RNA 3'end Desthiobiotinylation Kit (#20163, Thermo Fisher Scientific) was applied to label RNAs with desthiobiotinylate. Pierce^TM^ Magnetic RNA‐protein Pull Down Kit (#20164, Thermo Fisher Scientific) was used to implement an RNA‐protein pull‐down assay. Briefly, 2 μg labeled sense and anti‐sense probes were incubated for 6 h with streptavidin magnetic beads in 500 μL binding buffer at 4°C with rotation. Subsequently, the biotinylated lncRNA streptavidin magnetic beads mixture was cultured for 6 h with the cell lysates at 4°C with rotation. Part of the cell lysates were used as an input. Lastly, a western blot assay was conducted to analyze the protein after eluting the RNA‐binding protein.

### 2.17 Statistical analysis

All data were presented as mean ± standard deviation (SD), and GraphPad Prism 8.0 software was used to process data. One‐way analysis of variance with Tukey's post hoc test was performed for data comparison. A *p*‐value of less than 0.05 was regarded as statistically significant.

## 3 RESULTS

### 3.1 LncRNA *Sngh12* is highly expressed in human and mouse liver fibrotic tissues

**FIGURE 1:**
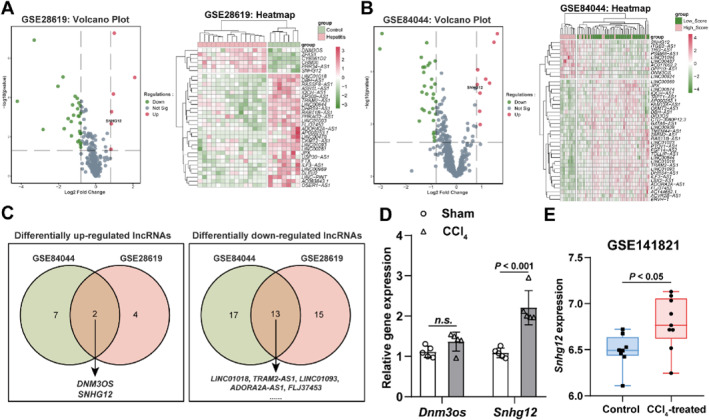
LncRNA *Snhg12* exhibits high expression level within liver fibrotic tissue samples based on microarray‐based analysis. (A), Volcano and heat maps of GSE28619 dataset; control: *n* = 5, hepatitis: *n* = 15. (B), Volcano and heat maps of GSE84044 dataset; low score patients: *n* = 20, high score patients: *n* = 37. (C), Venn screening of GSE28619 and GSE84044 for co‐differentially expressed lncRNAs in liver fibrosis. (D), qRT‐PCR was conducted to detect *Dnm3os* and *Snhg12* expression within mouse liver tissue samples; *n* = 5. (E), Expression of *Snhg12* in GSE141821; control (olive oil) mice: *n* = 8, CCl_4_‐treated mice: *n* = 9. n.s, *p* < 0.05.

DEGs in liver fibrotic tissues were first searched from the GEO liver fibrosis‐related data microarrays. In GSE28619, there were 6 up‐regulated and 28 down‐regulated lncRNAs in alcoholic hepatitis patients' liver tissues than normal controls (Figure 1A). In GSE84044, compared to liver tissues of hepatitis B patients without fibrosis, there were 9 up‐regulated and 30 down‐regulated lncRNAs in liver fibrotic tissues (Figure 1B). The DEGs from these two microarrays were taken for intersection, and a total of 2 up‐expressed lncRNAs (*DNM3OS* and *SNHG12*) and 13 down‐expressed lncRNAs (Figure 1C) were obtained. This study aims to find underlying therapeutic targets for hepatic fibrosis; therefore, the 2 up‐regulated expressed lncRNAs were selected for subsequent studies.

*Dnm3os* and *Snhg12* expression levels within liver tissue samples of the CCl_4_‐induced mouse liver fibrosis model were evaluated using qRT‐PCR, indicating that the expression of *Snhg12* showed to be considerably higher within the liver tissue samples of CCl_4_‐induced mice in contrast to sham‐operated mice (Figure 1D). Meanwhile, *Snhg12* expression in GSE141821 microarrays was further examined, and *Snhg12* expression was notably elevated within the liver tissue samples of CCl_4_‐induced mice in contrast to control mice (Figure 1E). It is summarized that lncRNA *Snhg12* exhibits high expression level within liver fibrotic tissues.

### 3.2 Knockdown of lncRNA *Snhg12* inhibits liver fibrosis in mice

**FIGURE 2:**
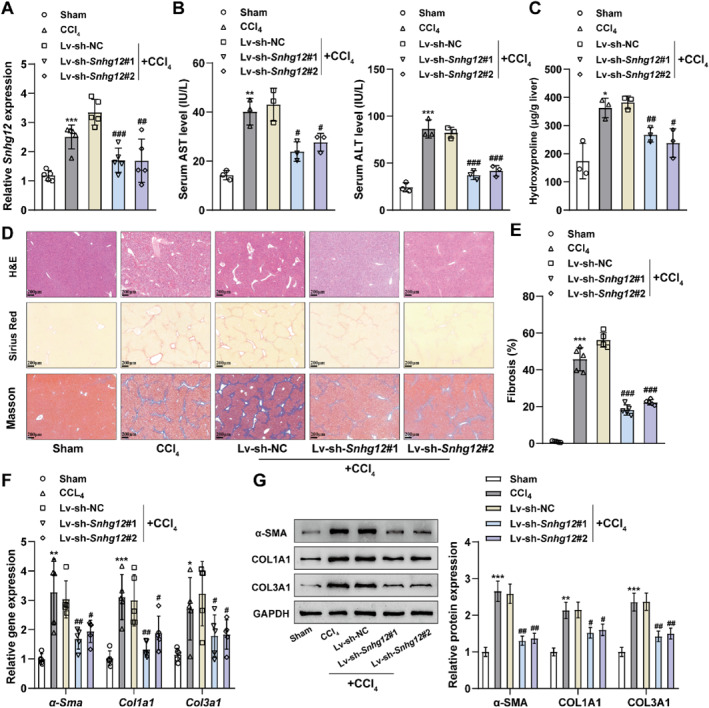
Knockdown of lncRNA *Snhg12* inhibits liver fibrosis in mice. Knockdown of *Snhg12* expression in the liver using *Snhg12* knockdown lentivirus by tail vein injection within a CCl_4_‐induced liver fibrosis mouse model. (A), qRT‐PCR was conducted to test *Snhg1*2 mRNA expression within mouse liver tissues. (B), AST and ALT contents in mouse serum were measured by AST and ALT content assay kits. (C), Hydroxyproline content assay kit was employed to determine hydroxyproline content in mouse liver tissues. (D), Pathologic damage and fibrosis of liver tissues were observed by H&E, Sirius Red, and Masson stainings. (E), Quantification of fibrosis in mouse liver tissues. (F, G), The expression levels of fibrosis‐related proteins *α‐Sma*, *Col1a1*, and *Col3a1* mRNA and protein levels within mouse liver tissues were determined using qRT‐PCR and western blot. *N* = 3. *p < 0.05, *p < 0.01, ***p < 0.001 compared to Sham group; #*p* < 0.05, ##*p* < 0.01, ###*p* < 0.001 compared to Lv‐sh‐NC group. AST, aspartate aminotransferase; ALT, alanine aminotransferase.

Given that lncRNA *Snhg12* expression showed to be increased within liver fibrotic tissue samples, lncRNA *Snhg12* knockdown subsequently explored its function in a liver fibrosis mouse model. CCl_4_‐treated liver fibrosis mice were infected with Lv‐sh‐*Snhg12*#1 and Lv‐sh‐*Snhg12*#2 knockdown lentiviruses, as confirmed by qRT‐PCR, revealing that Snhg12 knockdown lentiviruses significantly reduced *Snhg12* expression within the mouse liver tissues (Figure 2A). The serum levels of AST and ALT showed to be dramatically increased within CCl_4_‐induced mice compared to sham‐operated mice (Figure 2B), and the hydroxyproline levels in the liver tissues showed to be higher (Figure 2C). The pathological structural changes in mouse liver were observed by Hematoxylin and eosin, Sirius Red and Masson stainings. The results suggested that sham‐operated mice had a clear structure of liver lobules, well‐arranged hepatocyte cords, no degeneration, necrosis, fibrosis, inflammatory cell infiltration and lipid accumulation; whereas, CCl_4_‐treated mice had enlarged fibrosis in the portal area, obvious lipid accumulation, fiberformation, the disappearance of the lobule structure, and the formation of pseudo lobe individually (Figure 2D–E).

Activated HSCs are key to the formation and progression of liver fibrosis and are marked by *α*‐SMA expression, and the production of a large amount of extracellular matrix (ECM) shows to be dominated by collagen type I (COLLAGEN I, COL1A1), and collagen type III (COLLAGEN III, COL3A1).^22^ Therefore, qRT‐PCR and western blot were carried out to further examine the expression levels of fibrosis‐related proteins within mouse liver tissue samples. *α‐Sma*, *Col1a1*, *and Col3a1* mRNA and protein expression levels were notably elevated within liver tissue samples of CCl_4_‐treated mice compared to sham‐operated mice (Figure 2F–G). Compared to the Lv‐sh‐NC group, both the Lv‐sh‐*Snhg12*#1 and Lv‐sh‐*Snhg12*#2 groups were able to reduce AST and ALT serum levels and hydroxyproline levels in liver tissues and suppress the inflammatory and fibrotic processes of the liver as well as *α‐Sma*, *Col1a1*, *and Col3a1* mRNA and protein levels within mouse liver tissue samples (Figure 2A–G). The aforesaid results indicate that CCl_4_ could successfully induce a mouse model of liver fibrosis, while interference with *Snhg12* expression partially reverses the process of liver fibrosis in mice.

### 3.3 LncRNA *Snhg12* is up‐regulated in activated mHSCs

**FIGURE 3:**
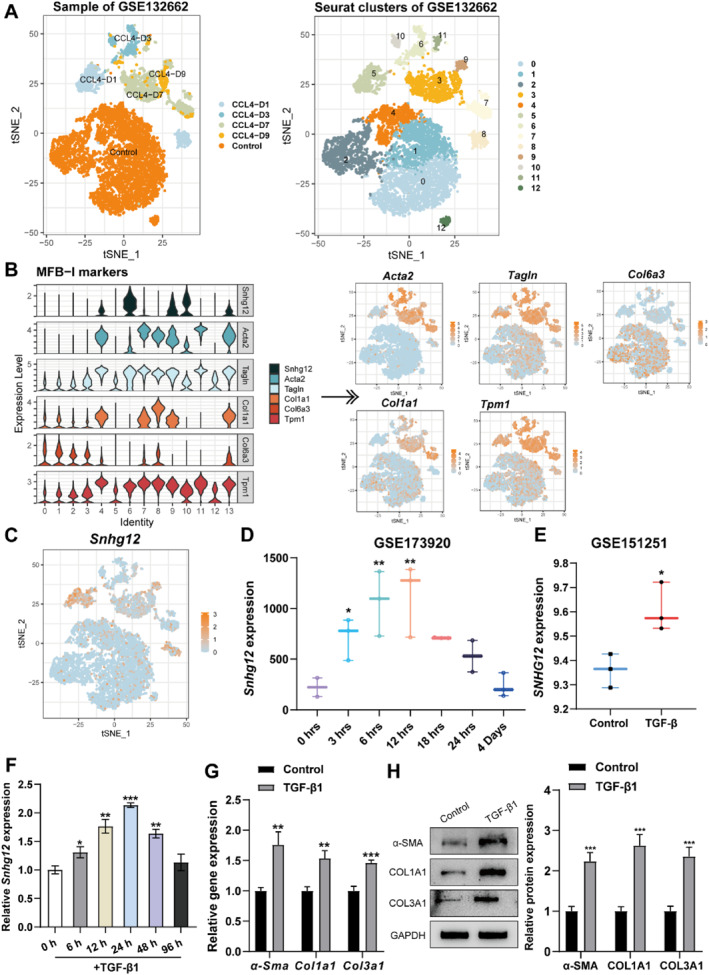
LncRNA *Snhg12* is up‐regulated in activated HSCs. (A), UMAP dimensional reduction visualization of cellular subpopulations in liver tissues in GSE132662. (B), Violin plots demonstrating the relative gene expression of activation markers; feature plots showing the relative gene expression strength of selected MFB I marker genes. (C), Feature plots showing the *Snhg12* gene expression strength in GSE132662. (D), Changes in transcript levels of *Snhg12* in GSE173920 analyzed by Illumina NovaSeq 6000 S Prime (SP) sequencing at various hours after mHSCs activation; *n* = 2 or 3. (E) lncRNA *SNHG12* expression within GSE151251 showed to be increased in TGF‐β1‐treated human HSCs; *n* = 3. (F), After 0‐h, 6‐h, 12‐h, 24‐h, 48‐h, and 96‐h treating mHSCs with 25 ng/mL TGF‐β1, qRT‐PCR was carried out to detect *Snhg12* expression in mHSCs. (G, H), *α‐Sma*, *Col1a1*, and *Col3a1* mRNA and protein expression within TGF‐β1 treated mHSCs for 24 h were tested using qRT‐PCR (G) and western blot (H) assays. *N* = 3. *p < 0.05, **p < 0.01, ***p < 0.001 compared to 0 h group or control group. MFB, myofibroblasts; mHSCs, mouse hepatic stellate cells; UMAP, Uniform manifold approximation and projection.

The key to liver fibrosis is HSC activation. When the liver is injured, HSCs are activated from quiescent retinol storage cells to myofibroblasts (MFB), resulting in liver fibrogenesis and promoting its development.^23^, ^24^ Therefore, the effect of *Snhg12* on mHSC activation was further investigated. GSE132662 included single‐cell gene expression data of mouse HSCs in different time‐activated states at rest and CCL_4_ treatment,^13^ which contains the expression data matrix of 14,939 genes from 8547 cells, and after data filtering as well as dimensionality reduction, 14 cellular subpopulations (tSNE projections) were obtained (Figure 3A). According to the markers of each cluster provided by Krenkel et al.,^13^ cluster 4, 6, 7, 8, 10, and 13 are the early stage of activated MFB, termed MFB I, and their markers include *α‐Sma* (*Acta2*), *Tagln*, *Col1a1*, *Tpm1* and *Col6a3* (Figure 3B). According to the cell sorting markers, it was observed that lncRNA *Snhg12* expression was co‐expressed in clusters 4, 5, 6, 10, and 13, suggesting that *Snhg12* may function in the early HSC activation and their myofibroblast differentiation (Figure 3C). GSE173920 contained data on transcriptional changes at various hours after mHSCs activation,^14^ and the analysis showed that gene expression of Snhg12 increased rapidly at 3 h after activation, peaked at 6–12 h, and then gradually declined and returned to normal levels on day 4 (Figure 3D). Moreover, the expression level of *SNHG12* was further analyzed using GSE151251, which revealed that *SNHG12* was significantly increased after TGF‐*β* treatment on human HSCs (Figure 3E). These microarray‐based analysis results unveiled that Snhg12 may be involved in mHSC activation. Consistent with microarray‐based analysis, it has been revealed by qRT‐PCR that *Snhg12* expression level was notably elevated after TGF‐β1 treatment with different times in mHSCs, which reached the highest degree of elevation at 24 h of TGF‐β1 treatment (Figure 3F). Hence, 24 h of TGF‐β1 treatment in mHSCs was applied for the future experiments. The levels of fibrosis‐associated proteins *α‐Sma*, *Col1a1*, *and Col3a1* mRNA and protein in mHSCs were examined by qRT‐PCR and western blot using TGF‐β1‐treated mHSCs. The findings suggested that TGF‐β1 markedly enhanced the expression levels of fibrosis‐associated proteins in mHSCs (Figure 3G–H). Taken together, *Snhg1*2 might contribute to mHSC activation.

### 3.4 lncRNA *Snhg12* knockdown impedes TGF‐β1 induced mHSCs proliferation and activation

**FIGURE 4:**
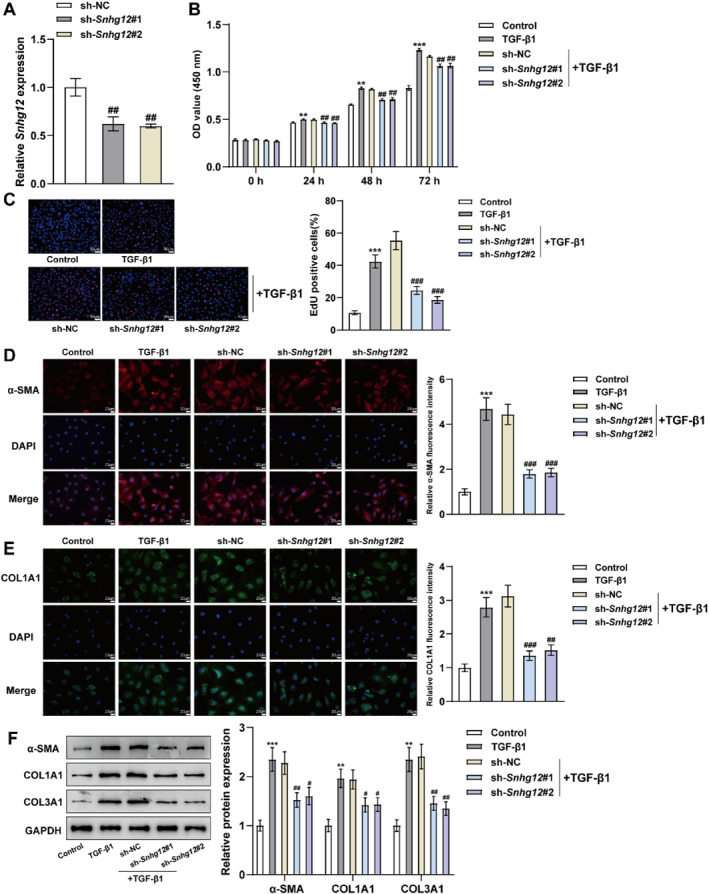
Knockdown of lncRNA *Snhg12* impedes TGF‐β1‐induced mHSCs proliferation and activation. (A), mHSCs were transfected with sh‐*Snhg12*#1&2 knockdown plasmid and its control vector plasmid sh‐NC, respectively. Following 48 h of transfection, cells were harvested, and the transfection interference efficiency was determined by qRT‐PCR. (B–F), After 24‐h treating mHSCs with 25 ng/mL TGF‐β1: (B & C). mHSC cell proliferation was measured using CCK‐8 (B) and EdU (C) assays. (D), Fluorescence microscopy was conducted to observe *α*‐SMA and COL1A1 expression level in mHSCs. (E) *α*‐SMA, COL1A1, and COL3A1 protein levels within mHSCs were evaluated using Western blot. *N* = 3, **p < 0.01, ***p < 0.001 compared to Control group; ##*p* < 0.01, ###*p* < 0.001 compared to sh‐NC group. α‐SMA, α‐smooth muscle action; mHSCs, mouse hepatic stellate cells.

Given that the expression of lncRNA *Snhg12* was increased within mouse liver fibrosis tissue samples and TGF‐β1‐induced mHSC cell models, the function of *Snhg12* in mHSCs proliferation and activation was investigated by knocking down lncRNA *Snhg12*. LncRNA *Snhg12* knockdown efficiency was verified using qRT‐PCR, indicating that both sh‐*Snhg12*#1 and 2 remarkably reduced *Snhg12* expression in the cells (Figure 4A). CCK‐8 and EdU assays were performed to estimate cell proliferation after stimulation of mHSCs using 25 ng/mL TGF‐β1, and the findings were consistent with the expectation that TGF‐β1 could notably boost the proliferation of HSCs (Figure 4B–C). Meanwhile, as shown by immunofluorescence and western blot assays, TGF‐β1 advanced the expression levels of fibrogenesis‐associated proteins *α*‐SMA, COL1A1, and COL3A1 (Figure 4D–F). This result indicates that the mHSC cell fibrosis model induced by TGF‐β1 was constructed successfully. Furthermore, the knockdown of lncRNA *Snhg12* significantly neutralized the TGF‐β1‐induced enhancement in mHSCs proliferation and fibrosis‐related protein expression levels (Figure 4B–F). In conclusion, the proliferation and activation of mHSCs were markedly reduced by knocking down *Snhg12,* a lncRNA that was abnormally overexpressed in liver fibrosis.

### 3.5 LncRNA *Snhg12* directly binds IGFBP3 and enhances its stability

**FIGURE 5:**
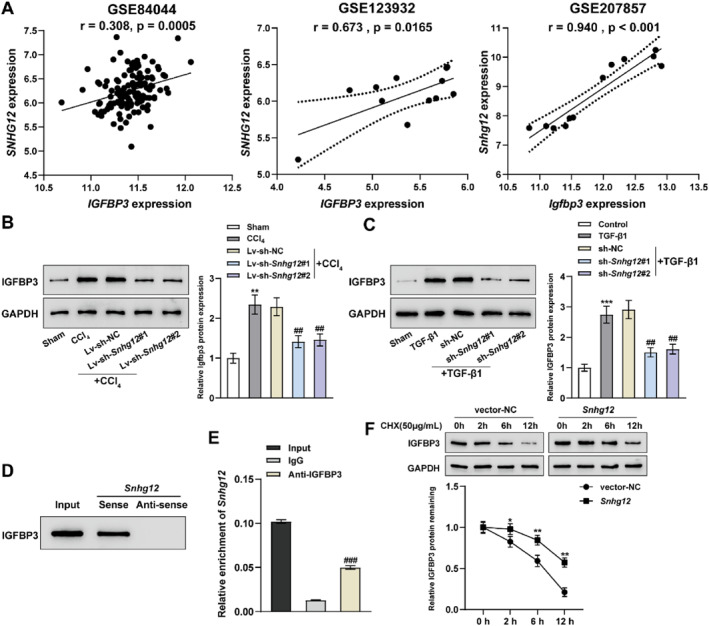
LncRNA *Snhg12* directly binds IGFBP3 and enhances its stability. (A), The expression correlation of lncRNA *Snhg12* to *Igfbp3* in GSE84044, GSE123932, and GSE207857, respectively, was verified using Pearson test. (B), IGFBP3 protein levels in liver tissues were detected with Western blot after using *Snhg12* knockdown lentiviral infection within a CCl_4_‐treated liver fibrosis mouse model. (C), IGFBP3 protein levels in cells were detected with Western blot after using *Snhg12* interference plasmid transfection in TGF‐β1‐induced mHSCs. (D, E), RNA pull down (D) and RIP assays (E) were performed to validate the binding efficiency between lncRNA *Snhg12* and IGFBP3 protein. (F), IGFBP3 protein expression levels were assessed by Western blot was carried out to assess IGFBP3 levels following 48‐h transfection of mHSCs with the *Snhg12* overexpression plasmid and following 0‐, 2‐, 4‐, and 6‐h treatments with cycloheximide (50 μg/mL). *N* = 3, **p < 0.01, ***p < 0.001 compared to normal control; ##*p* < 0.01 compared to sh‐NC group. mHSCs, mouse hepatic stellate cells.

IGFBP3 is one of the important carriers of IGF in blood circulation. It has been reported that IGFBP3 is a marker of culture‐induced HSC activation,^25^ and could be used as a diagnostic and therapeutic target for alcoholic fatty liver and liver fibrosis.^26^ Notably, lncRNA *Snhg12* and *Igfbp3* expression levels exhibited a positive correlation within human and mouse liver fibrosis expression datasets (GSE84044, GSE123932, and GSE207857) (Figure 5A). Further analysis revealed that increased expression of Igfbp3 was also detected in mouse liver fibrotic tissues and activated mHSCs, and the knockdown of lncRNA *Snhg12* significantly suppressed IGFBP3 expression (Figure 5B–C). Taken together, lncRNA *Snhg12* positively regulates IGFBP3 expression. Moreover, the RNA‐Protein Interaction Prediction (RPISeq) database was used to predict the protein binding region of lncRNA *Snhg12* to IGFBP3. Binding was found to be likely in both humans and mice (Table S3 in Supporting Information S1). RNA pull‐down and RIP assays were conducted to examine the binding efficiency between lncRNA *Snhg12* and IGFBP3, suggesting that lncRNA *Snhg12* could directly target IGFBP3 (Figure 5D–E), and lncRNA *Snhg12* overexpression enhanced IGFBP3 protein stability (Figure 5F). It is suggested that increased expression of *Igfbp3* in mouse liver fibrotic tissues and mHSCs correlates with direct binding and enhanced stability of IGFBP3 by lncRNA *Snhg12*.

### 3.6 LncRNA *Snhg12*/*Igfbp3* advances TGF‐β1‐induced mHSCs proliferation and activation

**FIGURE 6:**
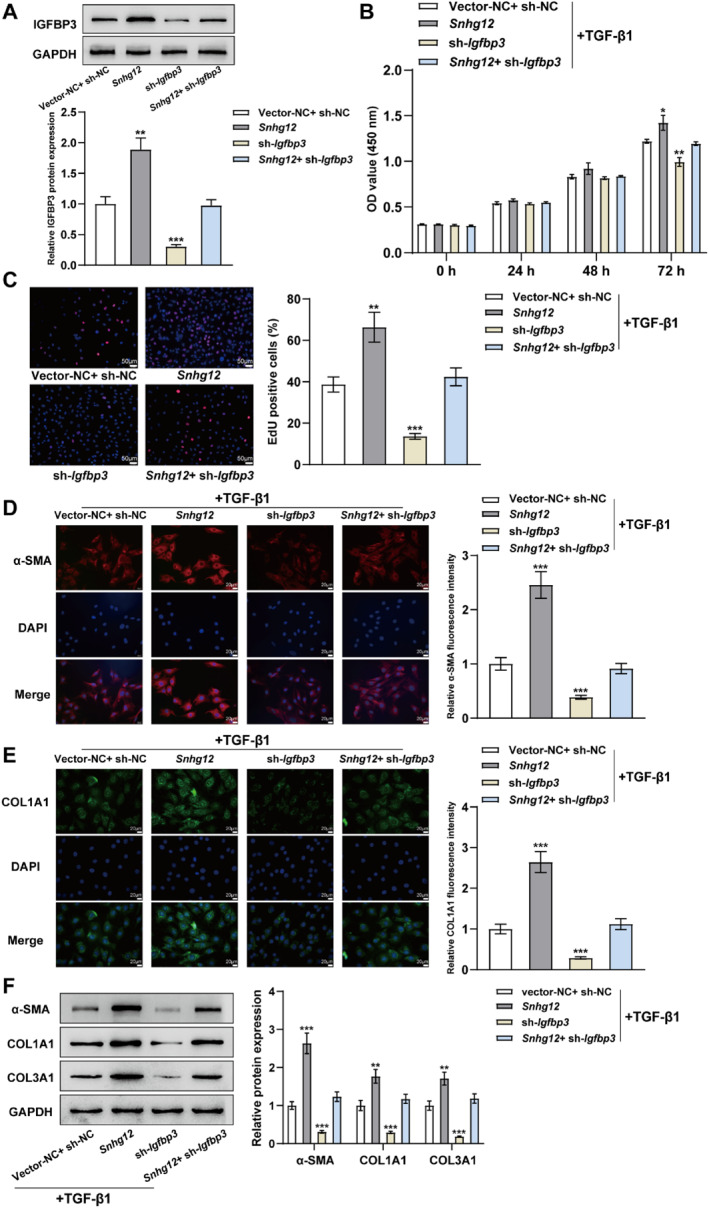
LncRNA *Snhg12*/*Igfbp3* boosts TGF‐β1‐induced mHSCs proliferation and activation. (A), After co‐transfection of mHSCs with *Snhg12* overexpression vector and/or *Igfbp3* knockdown vector for 48 h, IGFBP3 level in the cells was evaluated using Western blot. (B–F), mHSCs were cotransfected with *Snhg12* overexpression vector and/or *Igfbp3* knockdown vector, and 24 h after transfection, treatment with 25 ng/mL TGF‐β1 was continued for 24 h: B&C. The proliferation of mHSCs was evaluated using CCK‐8 (B) and EdU (C) assays. (D, E), Expression of *α*‐smooth muscle action (D) and COL1A1 (E) in mHSCs was observed by fluorescence microscopy. (E), Western blot was implemented to evaluate the levels of *α*‐SMA, COL1A1, and COL3A1 within mHSCs. *N* = 3, *p < 0.05, **p < 0.01, ***p < 0.001 compared to Vector‐NC + sh‐NC group. mHSCs, mouse hepatic stellate cells.

As mentioned above, lncRNA *Snhg12* promoted TGF‐β1‐induced mHSCs proliferation and activation, which is directly bound to Igfbp3 and enhanced its protein stability. Considering these findings, whether the lncRNA *Snhg12* fulfilled the above functions by interacting with IGFBP3 was investigated. The mHSCs were co‐transfected using *Snhg12* overexpression vector and/or *Igfbp3* knockdown vector, and after 24 h of transfection, the mHSCs were subjected to 24‐h treatment with 25 ng/mL TGF‐β1. The results of western blot demonstrated that the overexpression of *Snhg12* significantly up regulated the protein level of IGFBP3 (Figure 6A). According to the CCK8 and EdU assays, it was observed that the overexpression of *Snhg12* boosted mHSCs proliferation (Figure 6B–C). Immunofluorescence and western blot assays demonstrated that Snhg12 overexpression enhanced the levels of fibrosis‐associated proteins *α*‐SMA, COL1A1, and COL3A1 (Figure 6D–F). This contrasted with the function of knockdown of *Igfbp3* on mHSCs, whereas concomitant interference with *Igfbp3* partially reversed the promotion of IGFBP3 protein expression and mHSC activation by *Snhg12* overexpression. The effects of lncRNA *Snhg12*/*Igfbp3* on mHSC proliferation and activation without TGF‐β1 stimulation were also investigated (Figure S1 in Supporting Information S1). The overexpression of *Snhg12* or knockdown of *Igfbp3* had no obvious effects on mHSC cell viability and proliferation without TGF‐β1 stimulation (Figure S1A–B in Supporting Information S1). Moreover, there were no obvious changes for *α*‐SMA, COL1A1, and COL3A1 protein levels under *Snhg12* overexpression or *Igfbp3* knockdown in mHSCs without TGF‐β1 stimulation (Figure S1C in Supporting Information S1). Taken together, lncRNA *Snhg12* can promote TGF‐β1‐induced mHSCs proliferation and activation and subsequently advances the liver fibrosis process by up‐regulating IGFBP3 expression.

## 4 DISCUSSION

HSCs play a pivotal role in the progression of hepatic fibrosis. Kent et al.^27^ have found that HSCs are very closely related to ECM in injured livers, and it is proposed that activated HSCs have been identified as the main ECM source. Moreover, inducing HSC apoptosis or transformation to the quiescent phase can reverse the progression of hepatic fibrosis to some extent.^28^ The transdifferentiation of HSC from quiescent, vitamin A‐storing cells to proliferating, fibroblastic myofibroblasts and the secretion of *α*‐SMA, Col1a1, and Col3a1 are stimulated by various injuries; cytokines with pro‐fibrotic properties such as TGF‐β1 are now recognized as major drivers of liver fibrosis. However, the molecular mechanisms that regulate HSC proliferation are unclear to date. There is increasing evidence that lncRNAs serve as key regulatory molecules contributing to various biological activities. Herein, lncRNA *Snhg12* exhibited abnormally high expression within mouse liver fibrotic tissue samples and TGF‐β1‐treated mHSCs, and the knockdown of *Snhg12* retarded the development of liver fibrosis in mice by suppressing HSC proliferation and activation.

Current research on *SNHG12* is mainly focused on tumors. *SNHG12* is highly expressed in gastric carcinoma, non‐small cell lung cancer, and liver carcinoma, and is involved in tumor development and drug resistance, which is an underlying therapeutic target and biomarker for human cancers.^29^ For instance, *SNHG12* targets miR‐199a‐5p within hepatocellular carcinoma through a ceRNA mechanism, thereby regulating the MLK3/IkB‐α/NF‐kB signaling.^30^ In renal cell carcinoma and osteoarthritis, *SNHG12* has been demonstrated to modulate collagen expression including Col1a1 and Col2a1.^31^ Similarly, in this paper, *Snhg12* also regulated collagen deposition in the activated mHSCs. With regard to ceRNA, *SNHG12* has been revealed to bind to HuR protein to accelerate gastric cancer progression.^32^ In cardiovascular‐related studies, *SNHG12* could interact with DNA‐dependent protein kinase (DNA‐PK) and IGF2BP3 and play a protective role in atherosclerosis and angiogenic endothelial cells' response to ischemia.^33^, ^34^ This study concluded that *Snhg12* was positively associated with Igfbp3 expression in mHSCs and could interact with IGFBP3 to stabilize it, as predicted by bioinformatics analysis and experimentally verified.

As reported, IGFBP3 is a liver fibrosis progression‐associated protein; however, its role in liver fibrosis is currently inconclusive. For example, *IGFBP3* is a diagnostic and therapeutic target for alcoholic fatty liver and liver fibrosis.^26^ Moreover, the results of cellular experiments showed that *IGFBP3* was significantly up‐regulated during HSC activation; animal experiments revealed that silencing *IGFBP3* suppressed the activation of HSCs and the development of hepatic fibrosis.^25^ Nevertheless, Liu et al.^35^ reported a conflicting finding, which demonstrated that IGFBP‐3, DKK‐3, and DKK‐1 produced by amniotic membrane‐derived human amniotic membrane mesenchymal stem cells (hAMSCs) impede HSC activation and alleviate the mouse hepatic fibrosis by blocking the classical Wnt signaling. In this study, interference with *Igfbp3* markedly inhibited the activation of mHSCs, as evidenced by inhibition of proliferation and reduced ECM deposition. In contrast, overexpression of *Snhg12* could boost HSC proliferation and activation and exacerbate liver fibrosis by up‐regulating IGFBP3 expression. Multiple substance‐binding sites in the IGFBP3 structural domain^36^ contribute to the diversity of IGFBP3 biological functions. According to current research, the mechanism of its role in various diseases is categorized as IGF‐dependent and IGF‐independent, and, with different mechanisms, it plays the opposite role of promoting or alleviating disease in different diseases. Furthermore, different stages of HSC activation may also affect gene expression levels.^37^ Therefore, more studies are warranted to explore the effect of IGFBP3 on liver fibrosis and related molecular mechanisms.

In summary, this study underlines for the first time that lncRNA *Snhg12* expression is increased within liver fibrosis tissues of mice, and the *Snhg12*/IGFBP3 axis could promote liver fibrosis by promoting HSC proliferation and activation. *Snhg12* has been identified to be an underlying target for treating liver fibrosis.

## AUTHOR CONTRIBUTIONS

Conceptualization: Zheng Zhang. Investigation: Jinmao Liao, Qi Yuan, Lidan Luo and Xiaoxuan Hu. Resources: Lidan Luo and Xiaoxuan Hu. Validation: Lidan Luo and Xiaoxuan Hu. Writing: Jinmao Liao and Qi Yuan. Supervision: Zhenzhen Li and Zheng Zhang.

## CONFLICT OF INTEREST STATEMENT

The authors confirm that there are no conflicts of interest.

## ETHICS STATEMENT

The guidelines for the care and use of animals were approved by the Medicine Animal Welfare Committee of Hunan Provincial People's Hospital.

## Supporting information

Supporting Information S1

## Data Availability

The authors confirm that the data supporting the findings of this study are available within the article.

## References

[1] Parola, Maurizio , and Massimo Pinzani . 2019. “Liver Fibrosis: Pathophysiology, Pathogenetic Targets and Clinical Issues.” Molecular Aspects of Medicine 65: 37–55. 10.1016/j.mam.2018.09.002. 30213667

[2] Friedman, Scott L. , and Massimo Pinzani . 2022. “Hepatic Fibrosis 2022: Unmet Needs and a Blueprint for the Future.” Hepatology 75(2): 473–488. 10.1002/hep.32285. 34923653 PMC12179971

[3] Chen, Xing , and Li Huang . 2022. “Computational Model for ncRNA Research.” Briefings in Bioinformatics 23(6). 10.1093/bib/bbac472. 36274235

[4] Zhai, Wen , Xu Li , Shouzhen Wu , Yan Zhang , Huan Pang , and Wei Chen . 2015. “Microarray Expression Profile of lncRNAs and the Upregulated ASLNC04080 lncRNA in Human Endometrial Carcinoma.” International Journal of Oncology 46(5): 2125–2137. 10.3892/ijo.2015.2897. 25695231

[5] Liu, Yuenan , Gong Cheng , Ziwei Huang , Lin Bao , Jingchong Liu , Cheng Wang , Zhiyong Xiong , Lijie Zhou , Tianbo Xu , Di Liu , Hongmei Yang , Ke Chen , and Xiaoping Zhang . 2020. “Long Noncoding RNA SNHG12 Promotes Tumour Progression and Sunitinib Resistance by Upregulating CDCA3 in Renal Cell Carcinoma.” Cell Death & Disease 11(7): 515. 10.1038/s41419-020-2713-8. 32641718 PMC7343829

[6] Lu, Chenfei , Yutian Wei , Xiefeng Wang , Zhuoran Zhang , Jianxing Yin , Wentao Li , Lijiu Chen , Xiao Lyu , Zhumei Shi , Wei Yan , and Yongping You . 2020. “DNA‐methylation‐mediated Activating of lncRNA SNHG12 Promotes Temozolomide Resistance in Glioblastoma.” Molecular Cancer 19(1): 28. 10.1186/s12943-020-1137-5. 32039732 PMC7011291

[7] Huang, Yusheng , Lei Xia , Xiangwu Tan , Jingyi Zhang , Weiwei Zeng , Benxu Tan , Xian Yu , Wei Fang , and Zhenzhou Yang . 2022. “Molecular Mechanism of lncRNA SNHG12 in Immune Escape of Non‐small Cell Lung Cancer through the HuR/PD‐L1/usp8 axis.” Cellular and Molecular Biology Letters 27(1): 43. 10.1186/s11658-022-00343-7. 35658874 PMC9164758

[8] Wang, Shuqiang , Kun Chi , Di Wu , and Quan Hong . 2021. “Insulin‐Like Growth Factor Binding Proteins in Kidney Disease.” Frontiers in Pharmacology 12: 807119. 10.3389/fphar.2021.807119. 35002740 PMC8728008

[9] Martín, Ana Isabel , Teresa Priego , Álvaro Moreno‐Ruperez , Daniel González‐Hedström , Miriam Granado , and Asunción López‐Calderón . 2021. “IGF‐1 and IGFBP‐3 in Inflammatory Cachexia.” International Journal of Molecular Sciences 22(17): 9469. 10.3390/ijms22179469. 34502376 PMC8430490

[10] Ding, Ji‐Fei , He Sun , Kai Song , Yang Zhou , Bin Tu , K.‐Hu Shi , Dong Lu , S.‐Song Xu , and Hui Tao . 2023. “IGFBP3 Epigenetic Promotion Induced by METTL3 Boosts Cardiac Fibroblast Activation and Fibrosis.” European Journal of Pharmacology 942: 175494. 10.1016/j.ejphar.2023.175494. 36657656

[11] Affò, Silvia , Marlene Dominguez , Juan José Lozano , Pau Sancho‐Bru , Daniel Rodrigo‐Torres , Oriol Morales‐Ibanez , Montserrat Moreno , Cristina Millán , Aurora Loaeza‐del‐Castillo , José Altamirano , Juan Carlos García‐Pagán , Vicente Arroyo , Pere Ginès , Juan Caballería , Robert F. Schwabe , and Ramon Bataller . 2013. “Transcriptome Analysis Identifies TNF Superfamily Receptors as Potential Therapeutic Targets in Alcoholic Hepatitis.” Gut 62(3): 452–460. 10.1136/gutjnl-2011-301146. 22637703 PMC4064940

[12] Wang, Mingjie , Qiming Gong , Jiming Zhang , Liang Chen , Zhanqing Zhang , Lungen Lu , Demin Yu , Yue Han , Donghua Zhang , Peizhan Chen , X. Zhang , Zhenghong Yuan , Xiaonan Zhang , and X. Zhang . 2017. “Characterization of Gene Expression Profiles in HBV‐Related Liver Fibrosis Patients and Identification of ITGBL1 as a Key Regulator of Fibrogenesis.” Scientific Reports 7(1): 43446. 10.1038/srep43446. 28262670 PMC5337978

[13] Krenkel, Oliver , Jana Hundertmark , Thomas Ritz , Ralf Weiskirchen , and Frank Tacke . 2019. “Single Cell RNA Sequencing Identifies Subsets of Hepatic Stellate Cells and Myofibroblasts in Liver Fibrosis.” Cells 8(5): 503. 10.3390/cells8050503. 31137713 PMC6562512

[14] De Smet, Vincent , Nathalie Eysackers , Vincent Merens , Mina Kazemzadeh Dastjerd , Georg Halder , Stefaan Verhulst , Inge Mannaerts , and Leo A. van Grunsven . 2021. “Initiation of Hepatic Stellate Cell Activation Extends into Chronic Liver Disease.” Cell Death & Disease 12: 1110. 10.1038/s41419-021-04377-1. 34839349 PMC8627507

[15] Xi, Ying , Ryan LaCanna , H.‐Yen Ma , E.‐Noah N’Diaye , Sarah Gierke , Patrick Caplazi , Meredith Sagolla , Zhiyu Huang , Laura Lucio , Alexander Arlantico , Surinder Jeet , Hans Brightbill , Claire Emson , Aaron Wong , Katrina B. Morshead , Daryle J. DePianto , Merone Roose‐Girma , Charles Yu , Lucinda Tam , Guiquan Jia , Thirumalai R. Ramalingam , Scot Marsters , Avi Ashkenazi , Si Hyun Kim , Ryan Kelly , Shuang Wu , Paul J. Wolters , Ariel E. Feldstein , Jason A. Vander Heiden , and Ning Ding . 2022. “A WISP1 Antibody Inhibits MRTF Signaling to Prevent the Progression of Established Liver Fibrosis.” Cell Metabolism 34(9): 1377–1393.e8. 10.1016/j.cmet.2022.07.009. 35987202

[16] Liao, Jinmao , Zheng Zhang , Qi Yuan , Lidan Luo , and Xiaoxuan Hu . 2022. “The Mouse Anxa6/miR‐9‐5p/Anxa2 axis Modulates TGF‐Β1‐Induced Mouse Hepatic Stellate Cell (mHSC) Activation and CCl(4)‐Caused Liver Fibrosis.” Toxicology Letters 362: 38–49. 10.1016/j.toxlet.2022.04.004. 35483553

[17] Yi, Jing , Li Li , Z.‐jun Yin , Y.‐yun Quan , R.‐rong Tan , S.‐long Chen , Ji‐rui Lang , Jiao Li , Jin Zeng , Yong Li , Zi‐jian Sun , and J.‐ning Zhao . 2023. “Polypeptide from Moschus Suppresses Lipopolysaccharide‐Induced Inflammation by Inhibiting NF‐κ B‐ROS/NLRP3 Pathway.” Chinese Journal of Integrative Medicine 29(10): 895–904. 10.1007/s11655-023-3598-z. 37542626

[18] Zhou, Xiaolong , Jiaoyang Lu , Ben Wu , and Zhen Guo . 2022. “HOXA11‐AS Facilitates the Proliferation, Cell Cycle Process and Migration of Keloid Fibroblasts through Sponging miR‐188‐5p to Regulate VEGFA.” Journal of Dermatological Science 106(2): 111–118. 10.1016/j.jdermsci.2022.04.004. 35491288

[19] Wu, Wantao , He Li , Zeyu Wang , Ziyu Dai , Xisong Liang , Peng Luo , Kun Liu , Hao Zhang , Nan Zhang , Shuyu Li , and Chi Zhang . 2024. “The Tertiary Lymphoid Structure‐Related Signature Identified PTGDS in Regulating PD‐L1 and Promoting the Proliferation and Migration of Glioblastoma.” Heliyon 10(1): e23915. 10.1016/j.heliyon.2023.e23915. 38205335 PMC10777022

[20] Li, J.‐Ming , Xianyu Li , Lawrence W. C. Chan , Ruinian Hu , Tian Zheng , Haojie Li , and Sijun Yang . 2023. “Lipotoxicity‐polarised Macrophage‐Derived Exosomes Regulate Mitochondrial Fitness through Miro1‐Mediated Mitophagy Inhibition and Contribute to Type 2 Diabetes Development in Mice.” Diabetologia 66(12): 2368–2386. 10.1007/s00125-023-05992-7. 37615690

[21] Xu, Hongfa , Hao Wang , Wei Zhao , Sirui Fu , Yong Li , Wenjun Ni , Yongjie Xin , Wei Li , Chenzi Yang , Yanyan Bai , Meixiao Zhan , and Ligong Lu . 2020. “SUMO1 Modification of Methyltransferase‐like 3 Promotes Tumor Progression via Regulating Snail mRNA Homeostasis in Hepatocellular Carcinoma.” Theranostics 10(13): 5671–5686. 10.7150/thno.42539. 32483411 PMC7254988

[22] Khanam, Arshi , Paul G. Saleeb , and Shyam Kottilil . 2021. “Pathophysiology and Treatment Options for Hepatic Fibrosis: Can it Be Completely Cured?” Cells 10(5): 1097. 10.3390/cells10051097. 34064375 PMC8147843

[23] Zhang, Mengfan , Sandra Serna‐Salas , Turtushikh Damba , Michaela Borghesan , Marco Demaria , and Han Moshage . 2021. “Hepatic Stellate Cell Senescence in Liver Fibrosis: Characteristics, Mechanisms and Perspectives.” Mechanism of Ageing and Development 199: 111572. 10.1016/j.mad.2021.111572. 34536446

[24] Trivedi, Parth , Shuang Wang , and Scott L. Friedman . 2021. “The Power of Plasticity‐Metabolic Regulation of Hepatic Stellate Cells.” Cell Metabolism 33(2): 242–257. 10.1016/j.cmet.2020.10.026. 33232666 PMC7858232

[25] Mannaerts, Inge , Ben Schroyen , Stefaan Verhulst , Leentje Van Lommel , Frans Schuit , Marc Nyssen , and Leo A. van Grunsven . 2013. “Gene Expression Profiling of Early Hepatic Stellate Cell Activation Reveals a Role for Igfbp3 in Cell Migration.” PLoS One 8(12): e84071. 10.1371/journal.pone.0084071. 24358328 PMC3866247

[26] Polyzos, Stergios A. , Nikolaos Perakakis , Chrysoula Boutari , Jannis Kountouras , Wael Ghaly , Athanasios D. Anastasilakis , Asterios Karagiannis , and Christos S. Mantzoros . 2020. “Targeted Analysis of Three Hormonal Systems Identifies Molecules Associated with the Presence and Severity of NAFLD.” The Journal of Cinical Endocrinology and Metabolism 105(3): dgz172–e400. 10.1210/clinem/dgz172. PMC7112980 31690932

[27] Kent, G. , S. Gay , T. Inouye , R. Bahu , O. T. Minick , and H. Popper . 1976. “Vitamin A‐Containing Lipocytes and Formation of Type III Collagen in Liver Injury.” Proceedings of the National Academy of Sciences of the U S A 73(10): 3719–3722. 10.1073/pnas.73.10.3719. PMC431190 1068482

[28] Kamm, Dakota R. , and Kyle S. McCommis . 2022. “Hepatic Stellate Cells in Physiology and Pathology.” The Journal of Physiology 600(8): 1825–1837. 10.1113/jp281061. 35307840 PMC9012702

[29] Tamang, Suraksha , Varnali Acharya , Deepronil Roy , Rinka Sharma , Apeksha Aryaa , Uttam Sharma , Akanksha Khandelwal , Hridayesh Prakash , Karen M. Vasquez , and Aklank Jain . 2019. “SNHG12: An LncRNA as a Potential Therapeutic Target and Biomarker for Human Cancer.” Frontiers in Oncology 9: 901. 10.3389/fonc.2019.00901. 31620362 PMC6759952

[30] Lan, Tian , Weijie Ma , Zhenfei Hong , Long Wu , Xi Chen , and Yufeng Yuan . 2017. “Long Non‐coding RNA Small Nucleolar RNA Host Gene 12 (SNHG12) Promotes Tumorigenesis and Metastasis by Targeting miR‐199a/b‐5p in Hepatocellular Carcinoma.” Journal of Experimental and Clinical Cancer Research 36(1): 11. 10.1186/s13046-016-0486-9. 28073380 PMC5223416

[31] INVALID CITATION [24, 25]

[32] Zhang, Tianqi , Maneesh Kumarsing Beeharry , Yanan Zheng , Zhenqiang Wang , Jianfang Li , Zhenggang Zhu , and Chen Li . 2021. “Long Noncoding RNA SNHG12 Promotes Gastric Cancer Proliferation by Binding to HuR and Stabilizing YWHAZ Expression through the AKT/GSK‐3β Pathway.” Frontiers in Oncology 11: 645832. 10.3389/fonc.2021.645832. 34195070 PMC8236831

[33] Gross, David A. , Henry S. Cheng , Rulin Zhuang , Michael G. McCoy , Daniel Pérez‐Cremades , Zachary Salyers , A. K. M. Khyrul Wara , Stefan Haemmig , Terence E. Ryan , and Mark W. Feinberg . 2022. “Deficiency of lncRNA SNHG12 Impairs Ischemic Limb Neovascularization by Altering an Endothelial Cell Cycle Pathway.” JCI insight 7(1). 10.1172/jci.insight.150761. PMC8765056 34793334

[34] Haemmig, Stefan , Dafeng Yang , Xinghui Sun , Debapria Das , Siavash Ghaffari , Roberto Molinaro , Lei Chen , Yihuan Deng , Dan Freeman , Norman Moullan , Yevgenia Tesmenitsky , A. K. M. Khyrul Wara , Viorel Simion , Eugenia Shvartz , James F. Lee , Tianlun Yang , Galina Sukova , Jarrod A. Marto , Peter H. Stone , Warren L. Lee , Johan Auwerx , Peter Libby , and Mark W. Feinberg . 2020. “Long Noncoding RNA SNHG12 Integrates a DNA‐PK‐Mediated DNA Damage Response and Vascular Senescence.” Science Translational Medicine 12(531). 10.1126/scitranslmed.aaw1868. 32075942

[35] Liu, Q.‐Wen , Y.‐Min Ying , J.‐Xin Zhou , W.‐Jie Zhang , Z.‐xiao Liu , B.‐Bing Jia , H.‐Cheng Gu , C.‐Yu Zhao , X.‐Hui Guan , Ke‐Yu Deng , and H.‐Bo Xin . 2022. “Human Amniotic Mesenchymal Stem Cells‐Derived IGFBP‐3, DKK‐3, and DKK‐1 Attenuate Liver Fibrosis through Inhibiting Hepatic Stellate Cell Activation by Blocking Wnt/β‐Catenin Signaling Pathway in Mice.” Stem Cell Research & Therapy 13(1): 224. 10.1186/s13287-022-02906-z. 35659360 PMC9166579

[36] Ranke, Michael B . 2015. “Insulin‐like Growth Factor Binding‐Protein‐3 (IGFBP‐3).” Best Practice & Research Clinical Endocrinology & Metabolism 29(5): 701–711. 10.1016/j.beem.2015.06.003. 26522455

[37] Yang, Wu , Hao He , Tongtong Wang , Nan Su , Feng Zhang , Kai Jiang , Jing Zhu , Chonghe Zhang , Kongyan Niu , Luyue Wang , Xiaodong Yuan , Nan Liu , Lingjie Li , Wu Wei , and Junhao Hu . 2021. “Single‐Cell Transcriptomic Analysis Reveals a Hepatic Stellate Cell‐Activation Roadmap and Myofibroblast Origin during Liver Fibrosis in Mice.” Hepatology 74(5): 2774–2790. 10.1002/hep.31987. 34089528 PMC8597108

