# Supplementary material for: LncRNA Snhg12/IGFBP3 axis is involved in liver fibrosis by promoting the proliferation and activation of mouse hepatic stellate cells
Source: J Cell Commun Signal. 2024 May 28;18(2):e12033. doi: 10.1002/ccs3.12033 (PMC11208121; doi:10.1002/ccs3.12033)
Supplement: Supplementary file 1 — Supporting Information S1 [file CCS3-18-e12033-s001.docx]

**Supplementary Materials**


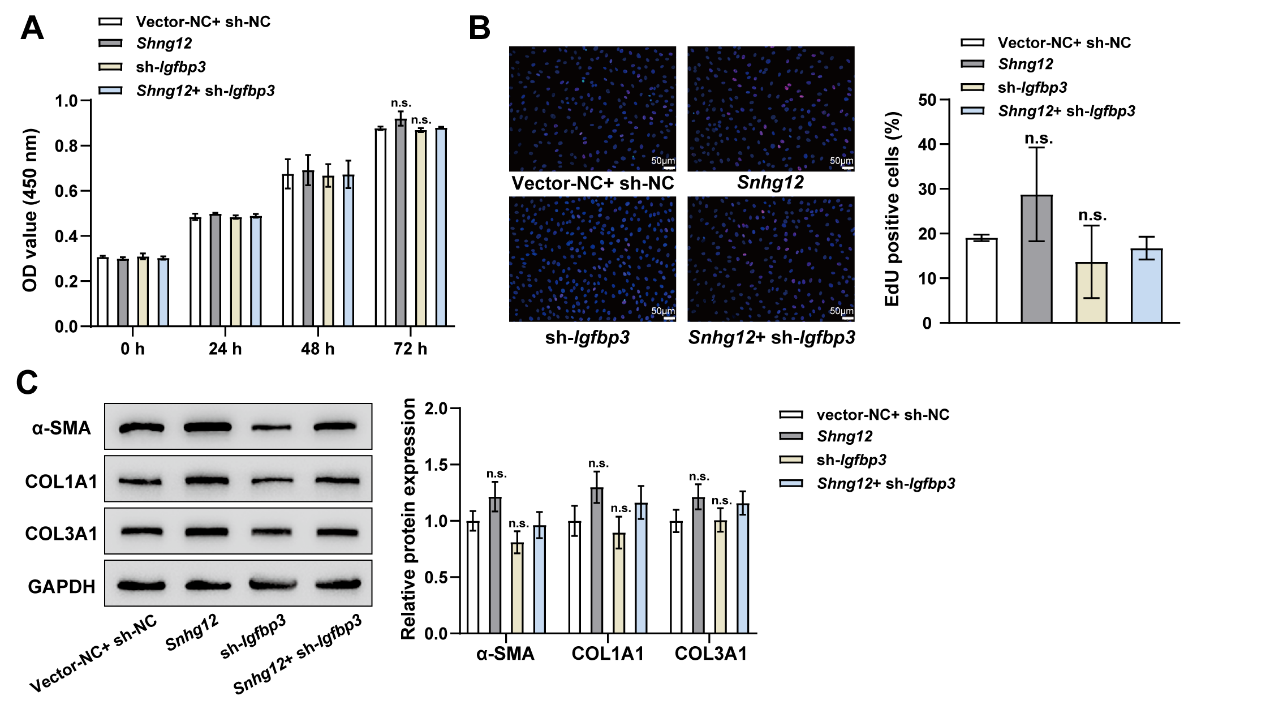


**Figure S1.** Effects of lncRNA *Snhg12*/*Igfbp3* on mHSCs proliferation and activation under without TGF-β1 stimulation. (A-C) mHSCs were transfected with Snhg12 overexpression vector and/or Igfbp3 knockdown vector for 48 h, and then detected for (A) cell viability by CCK-8 assay; (B) cell proliferation by EdU assay; (C) the protein levels of α-SMA, COL1A1, and COL3A1 by western blot assay. N = 3.

**Table S1. The primer sequence for PCR and vector construction**

| **Name** |  | **Sequence (5’-3’)** |
| --- | --- | --- |
| *Dnm3os* | Forward | GGCTGATTGTATCTGTCCCTGAG |
|  | Reverse | GCTTTCTTTCAATGGCTGGTG |
| *Snhg12* | Forward | GTCCAAGGAAGCACGGGTTAT |
|  | Reverse | TCTTCTGGTCTCCCTCCTCACA |
| *α-SMA* | Forward | CCCAGACATCAGGGAGTAATGG |
|  | Reverse | TCTATCGGATACTTCAGCGTCA |
| *Col1a1* | Forward | GCTCCTCTTAGGGGCCACT |
|  | Reverse | ATTGGGGACCCTTAGGCCAT |
| *Col3a1* | Forward | CAGGACCTAAGGGCGAAGATG |
|  | Reverse | TCCGGGCATACCCCGTATC |
| *Gapdh* | Forward | AGGTCGGTGTGAACGGATTTG |
|  | Reverse | TGTAGACCATGTAGTTGAGGTCA |
| *Snhg12*-RIP | Forward | GTCCAAGGAAGCACGGGTTAT |
|  | Reverse | TCTTCTGGTCTCCCTCCTCACA |
| sh-*Snhg12*#1 | Forward | GATCCGCCTTGTACTTCTACCCAACGCTCGAGCGTTGGGTAGAAGTACAAGGCTTTTTG |
|  | Reverse | AATTCAAAAAGCCTTGTACTTCTACCCAACGCTCGAGCGTTGGGTAGAAGTACAAGGCG |
| sh-*Snhg12*#2 | Forward | GATCCGGACAACGATACAGCAGAAGGCTCGAGCCTTCTGCTGTATCGTTGTCCTTTTTG |
|  | Reverse | AATTCAAAAAGGACAACGATACAGCAGAAGGCTCGAGCCTTCTGCTGTATCGTTGTCCG |
| sh-NC | Forward | GATCCGCAGATGAAGGCACGGTCACGCTCGAGCGTGACCGTGCCTTCATCTGCTTTTTG |
|  | Reverse | AATTCAAAAAGCAGATGAAGGCACGGTCACGCTCGAGCGTGACCGTGCCTTCATCTGCG |
| *Snhg12* overexpression | Forward | ctaccggactcagatctcgagTTTCTCGCTTCATCCGCG |
|  | Reverse | gtaccgtcgactgcagaattcCACTTTCCAAAAGTCTTTATTGAACC |
| Sh-*Igfbp3* | Forward | GATCCGAATCATCTGAAGTTCCTCAACTCGAGTTGAGGAACTTCAGATGATTCTTTTTG |
|  | Reverse | AATTCAAAAAGAATCATCTGAAGTTCCTCAACTCGAGTTGAGGAACTTCAGATGATTCG |

**Table S2. The antibody information**

| **Antibody** | **Brand** | **Dilution ratio** |
| --- | --- | --- |
| α-SMA | 55135-1-AP Proteintech | 1:1000 (western blot) |
|  |  | 1:200（IF） |
| COL1A1 | 66761-1-Ig, Proteintech | 1:1000 (western blot) |
|  |  | 1:200（IF） |
| COL3A1 | ab7778，Abcam | 1:1000 (western blot) |
| IGFBP3 | Ab220429, Abcam | 1:1000 (western blot) |
|  |  | 1:30（RIP） |
| GAPDH | #92310, Cell Signaling Technology (CST) | 1:5000 (western blot) |
| Anti-mouse IgG, HRP-linked | #7076, CST | 1:5000 (western blot) |
| Anti-rabbit IgG, HRP-linked | #7074, CST | 1:5000 (western blot) |
| Goat-anti-rabbit IgG H&L (Alexa Fluor® 647) | ab150083，abcam | 1:2000（IF） |
| Goat-anti-mouse IgG H&L (Alexa Fluor® 488) (ab175473) | ab150113，abcam | 1:2000（IF） |

**Table S3. Prediction of protein binding region between lncRNA *SNHG12* and IGFBP3**

| **lncRNA**  **names** | **Length** | **Prediction using Random Forest (RF) classifier** | **Prediction using Support Vector Machine (SVM) classifier** |
| --- | --- | --- | --- |
| *SNHG12* (human) | 1-1383 bp (full length) | 0.7 | 0.95 |
|  | 1-500 bp | 0.65 | 0.93 |
|  | 501-1000 bp | 0.65 | 0.93 |
|  | 1001 - 1383 bp | 0.7 | 0.92 |
| *Snhg12* (mouse) | 1-606 bp (full length) | 0.85 | 0.76 |
